# A Treatise on Apoplexy

**Published:** 1873-04

**Authors:** 


*A Treatise on Apoplexy, Cerebral Hemorrhage, Cerebral Embolism, Cerebral Gout, Cerebral Rheumatism, and Epidemic Cerebro-Spinal Meningitis.* By JOHN A. LIDELL, A. M., M. D., New York: Wm. Wood & Co., 1873. Buffalo: H. H. Otis.

This work the author informs us, has grown from an article which he intended formerly to publish in *The American Journal of Medical Science*, but as his number of cases increased his clinical experience enlarged, and the article became too extended for publication in a Journal.

Impressed with the idea that the labor and time invested in collecting and recording so many cases, and the thoughts which he had endeavored to set forth in their preparation made them too valuable to be thrown aside, the author has placed them in the hands of physicians and students in book form. The history of, and observations made in sixty-two cases are given.

The authors' ideas as far as we can judge by a hasty review of the work, are fully up to the times, and the lessons drawn from so many cases can not fail of imparting instruction to the readers of his work.

